# Clinical, laboratory, and chest CT features of severe versus non-severe pediatric patients with COVID-19 infection among different age groups

**DOI:** 10.1186/s12879-021-06283-5

**Published:** 2021-06-12

**Authors:** Meisam Hoseinyazdi, Saeid Esmaeilian, Reza Jahankhah, Arash Teimouri, Farzaneh Ghazi Sherbaf, Faranak Rafiee, Reza Jalli, Sedighe Hooshmandi

**Affiliations:** Medical Imaging research center, Shiraz University of Medical Sciences, Shiraz, Iran

**Keywords:** COVID-19, SARS-CoV-2, Pediatrics, CT, Children, Laboratory findings, Clinical symptoms

## Abstract

**Background:**

This study was performed with the intention of comparing the clinical, laboratory, and chest computed tomography (CT) findings between severe and non-severe patients as well as between different age groups composed of pediatric patients with confirmed COVID-19.

**Method:**

This study was carried out on a total of 53 confirmed COVID-19 pediatric patients who were hospitalized in Namazi and Ali Asghar Hospitals, Shiraz, Iran. The patients were divided into two severe (*n* = 27) and non-severe (*n* = 28) groups as well as into other three groups in terms of their age: aged less than two years, aged 3–12 years and 13–17 years. It should be noted that CT scans, laboratory, and clinical features were taken from all patients at the admission time. Abnormal chest CT in COVID-19 pneumonia was found to show one of the following findings: ground-glass opacities (GGO), bilateral involvement, peripheral and diffuse distribution.

**Result:**

Fever (79.2%) and dry cough (75.5%) were the most common clinical symptoms. Severe COVID-19 patients showed lymphocytosis, while the non-severe ones did not (*P* = 0.03). C-reactive protein (CRP) was shown to be significantly lower in patients aged less than two years than those aged 3–12 and 13–17 years (*P* = 0.01). It was shown also that O_2_ saturation experienced a significant increase as did patients’ age (*P* = 0.01). Severe patients had significantly higher CT abnormalities than non-severe patients (48.0% compared to 17.9%, respectively) (*P* = 0.02).

**Conclusion:**

Lymphocytosis and abnormal CT findings are among the factors most associated with COVID-19 severity. It was, moreover, showed that the severity of COVID-19, O_2_ saturation, and respiratory distress were improved as the age of confirmed COVID-19 pediatric patients increased.

## Background

An outbreak of unexplained viral infection called coronavirus 2019 (COVID-19) began in Wuhan, China in December 2019 [1, 2]. Since then, it has become a worldwide pandemic, causing infection in more than 28 million people (as of September 2020).

The number of affected children is on the rise according to recent studies. Meanwhile, pediatric patients are most commonly reported to show fever and cough [3, 4]. However, clinical, laboratory, and imaging findings regarding the pediatric population remain unclear. To date, the data suggests that children and young adults are less likely to suffer from more severe illness than adults [5]. Nevertheless, the recent increase in the reports about children having systemic inflammatory response, which require intensive care, has shown the need for prompt diagnosis [6]. Given that several cases with severe symptoms and even death have been observed among such patients, rapid and accurate diagnosis in the pediatric population is of the utmost significance [7].

Although the reverse transcriptase-polymerase chain reaction (RT-PCR) is recognized as a reference standard diagnostic test, computed tomography (CT) scan has turned into an important diagnostic tool, along with other clinical and laboratory features [8]. Bilateral ground-glass opacities (GGOs) with posterior and peripheral distribution in CT scan is known as the hallmark of COVID-19 pneumonia [9–11]. The main findings obtained from the abnormal CT scans of the pediatric population are peripherally located GGOs. Also, lower attenuation and a more localized extent of the GGOs are also reported in pediatric patients [12]. In some studies, CT scan findings were similar to those of the infected adult patients [12, 13]. Nevertheless, given the lower severity of COVID-19 pneumonia in pediatric patients, imaging findings, the pattern of involvement and the role of CT imaging in such patients are likely to be different from those which are commonly observed in adults.

In adults, COVID-19 manifestations range from asymptomatic infection to severe respiratory failure [14, 15]. Nonetheless, few studies have examined the severity of this disease among pediatrics aiming to make a distinction between severe and non-severe children with COVID-19 infection in clinical, laboratory, and radiological findings. Therefore, this study was conducted to identify the clinical and paraclinical characteristics of the pediatric population with COVID-19, compare different age groups of pediatric patients, as well as to run an analogy between severe and non-severe COVID-19 pediatric patients who were hospitalized in terms of their clinical, laboratory, and CT features.

## Methods

### Patients and study design

In this cross-sectional and multi-center study, a total of 53 pediatric patients (aged one month to 17 years old) with confirmed COVID-19 were admitted to the isolation wards of two hospitals composed of multispecialty healthcare university settings affiliated to Shiraz University of Medical Sciences between March 1, 2020 and May 30, 2020. The diagnosis was confirmed according to the interim guidance for novel coronavirus pneumonia published by the National Health Commission of the People’s Republic of China [16]. The inclusion criteria for the study were as follows: patients should be younger than 18 years of age, and show positive findings for COVID-19 via RT-PCR testing of respiratory secretions obtained through nasopharyngeal or oropharyngeal swab. The exclusion criteria were as follows: patients who were transferred to another hospital, were considered as uncooperative patients, lost to follow-up, had incomplete clinical and chest CT data and other lung infections. The patients included in the study were categorized based on the severity of their disease. Severity was defined in this study in accordance with the report presented by the New Coronavirus Pneumonia Prevention and Control Program (6th edition) published by the National Health Commission of China [17]: [1] Mild: no pneumonia in imaging (CT) [2]; Moderate: pneumonia diagnosed based on patients’ symptoms and imaging examination [3]; Severe: one of the following factors were observed: (i) respiratory rate was equal to or larger than 30/min; (ii) resting pulse oxygen saturation (SpO_2_) was equal to or smaller than 93%; (iii); partial pressure of oxygen (PaO_2_) was divided by the fraction of inspired oxygen (FiO_2_) and gave a value equal to or smaller than 300 mmHg (1 mmHg = 0.133 kPa); (iv) multiple pulmonary lobes showed more than 50% lesion progression in 24–48 h on imaging. Such patients were considered for anti-coagulants, dexamethasone and anti-biotics (clinically suspicious to bacterial infection) therapy, along with remdesivir and immunomodulator such as tocilizumab [4]; Critical, the last category, was defined if one of the following criteria are met: (i) the need for mechanical ventilation due to respiratory failure; (ii) shock; (iii) other complications requiring patents’ admission into intensive care unit (ICU). This category was taken into consideration for: anti-coagulant, steroids and anti-biotics (clinically suspicious to bacterial infection), remdesivir, tocilizumab and convalescent plasma treatment. Patients with mild or moderate disease were included in the “non-severe group” and severe or critical patients were categorized as the “severe group”. Moreover, patients were categorized in different age groups [18]: 1) patients under two years of age (Class I); 2) patients aged between 3 and 12 years old (Class II); and 3) patients aged between 13 and 17 years old (Class III). The study was carried out in compliance with the edicts of the *Declaration of Helsinki* and was approved by the Ethics Committee of Shiraz University of Medical Sciences, Shiraz, Iran (IR.SUMS.REC.1081).

### Data collection

In order to approve that patients were infected with the virus, reverse-transcription polymerase chain reaction (RT-PCR) was utilized to detect traces of SARS-CoV-2 nucleic acid in the patients. Also, to obtain RT-PCR samples, endotracheal aspirate, bronchoalveolar lavage, nasopharyngeal swab, or oropharyngeal swab were used, and moreover, chest CT was performed to diagnose pneumonia. Indeed, some samples were confirmed by RT-PCR at the admission time, while some other ones were then checked on stored samples due to lack of RT-PCR kits. Therefore, these patients were initially screened by chest CT. The obtained data was then reviewed and abstracted by two experienced radiologists. All the data about laboratory findings and other informations include: demographics, underlying medical conditions, clinical severity, the date for the onset of symptoms, the diagnosis date, the date hospitalization, and clinical outcome were extracted from electronic medical records. Information about underlying conditions were collected based on anamnestic data, according to the following categories: cardiovascular, hematologic, gastrointestinal and other comorbid diseases. The clinical manifestations such as cough, fever and dyspnea, laboratory findings including erythrocyte sedimentation rate (ESR), C-reactive protein (CRP), lymphocyte count and O_2_ saturation and chest CT images were all extracted from electronic medical records.

### CT scanning protocol

The following scanners were employed to scan all the patients: 16-MDCT Philips brilliance (Philips healthcare, United States), with 120–130 kvp, Ave 75 mAs, tubal current 103–147, pitch 1.1–1.2, slice thickness 5 mm, and reconstruction thickness 5 mm. Patients were scanned in the supine position and during a breath-hold after inhalation.

### Image viewing and evaluation

The analysis of all the CT images was performed based on the study by Zarei et al. [19] such as the patterns extracted from CT images grouping and classification of pleural changes. Also, the changes in bronchial were divided into two subcategories: air bronchogram (an air-filled image of bronchus in lung lesions) and bronchus distortion. It has recently been shown that the abnormal CT findings in COVID-19 include consolidation, GGO, bilateral involvement, peripheral and diffuse distribution [8, 20, 21].

### Statistical analysis

The SPSS Statistics 23.0 software (SPSS Inc., Chicago, Illinois, USA) was used to statistically analyze the data. The normality distribution of the data was examined using the Kolmogorov-Smirnov test. Continuous variables were presented as mean ± standard deviation (SD)/median and interquartile range (IQR) and were analyzed using independent t-test/Mann-Whitney U test. The categorical variables were presented by counts (percentage) and examined by χ2/Fisher’s exact tests. *P* < 0.05 was considered statistically significant.

## Results

### Basic and demographic findings

A comparison of basic clinical and laboratory characteristics of severe and non-severe patients is presented in Table 1. The patients’ mean age was 9.58 ± 5.35, ranging from two months to 17 years, and were mostly composed of females (31 females (58.5%) and 22 (41.5%) male). In the present study, a total of 25 and 28 patients were labeled as severe and non-severe patients, respectively. Patients in the severe group were younger (8.33 ± 5.51 years) than those of the non-severe group (10.69 ± 5.05 years). No significant age and sex differences were found between the severe and non-severe groups (*P* = 0.12, and *P* = 0.44, respectively). As shown in Table 2, after the patients were grouped based on their age, a number of seven, 27, and 19 subjects were placed in class I (≤2 years), class II (3–12 years), and class III (13–17), respectively. Five (71.4%), 14 (51.9%), and six (31.6%) patients showed severe signs in the age-specified class I, II, and III, respectively (*P* = 0.15). In addition, there was no significant sex difference between the three age-stratified groups (*P* = 0.27). Besides, the hospitalization process lasted considerably more in severe patients (7.16 ± 5.09 days) than in non-severe (3.78 ± 2.39 days) ones (*P* < 0.01).

**Table 1:** Comparison of clinical and laboratory features between severe and non-severe pediatric patients with COVID-19

	Total		Severe		Non severe		P- value
	N	Mean (SD)/percentage	N	Mean (SD)/percentage	N	Mean (SD)/percentage	
**Age,** year	53	9.58 (5.35)	25	8.33 (5.51)	28	10.69 (5.05)	0.12
**Sex**							
**Male**	22	41.5%	9	36.0%	13	46.4%	0.44
**Female**	31	58.5%	16	64.0%	15	53.6%	
**Disease onset to hospital admission duration**	53	3.0 (2.0–6.25)	25	4.0 (2.0–7.5)	28	3.0 (1.25–7.0)	0.38
**Hospital duration**	53	5.37 (4.22)	25	7.16 (5.09)	28	3.78 (2.39)	**0.01**
**ICU duration**	53	0.69 (2.46)	25	1.48 (3.45)	28	0.00 (0.00)	**0.01**
**ICU admission**							
**Yes**	6	11.3%	6	24.0%	0	0.0%	**0.01**
**No**	47	88.7%	19	76.0%	28	100.0%	
**Fever**							
**Yes**	42	79.2%	19	76.0%	23	82.1%	0.58
**No**	11	20.8%	6	24.0%	5	17.9%	
**Dry cough**							
**Yes**	40	75.5%	21	84.0%	19	67.9%	0.17
**No**	13	24.5%	4	16.0%	9	32.1%	
**Nasal congestion**							
**Yes**	5	9.4%	3	8.0%	2	10.7%	0.74
**No**	48	90.6%	23	92.0%	25	89.3%	
**Poor feeding**							
**Yes**	2	28.6%	1	20.0%	1	50.0%	0.43
**No**	5	71.4%	4	80.0%	1	50.0%	
**Body pain**							
**Yes**	14	30.4%	4	20.0%	10	38.5%	0.18
**No**	32	69.6%	16	80.0%	16	61.6%	
**Nausea**							
**Yes**	12	26.1%	6	30.0%	6	23.1%	0.60
**No**	34	73.9%	14	70.0%	20	76.9%	
**Diarrhea**							
**Yes**	5	9.4%	4	16.0%	1	3.6%	0.12
**No**	48	90.6%	21	84.0%	27	96.4%	
**Vomiting**							
**Yes**	11	20.8%	5	20.0%	6	21.4%	0.90
**No**	42	79.2%	20	80.0%	22	78.6%	
**Abdominal pain**							
**Yes**	3	6.5%	2	10.0%	1	3.8%	0.40
**No**	43	93.5%	18	90.0%	25	96.2%	
**Distress**							
**Yes**	23	43.4%	23	92.0%	0	0.0%	**< 0.01**
**No**	30	56.6%	2	8.0%	28	100.0%	
**Outcome**							
**Dead**	1	1.9%	1	4.0%	0	0.0%	0.28
**Alive**	52	98.1%	24	96.0%	28	100.0%	
**Leukocyte count,** × 10^9^/L	53	9232.08 (4755.19)	25	10,064.00 (4477.06)	28	8489.29 (4951.64)	0.10
**Leukopenia** (< 3.5 × 10^9^/L)							
**Yes**	3	5.7%	0	0.0%	3	10.7%	0.09
**No**	50	94.3%	25	100.0%	25	89.3%	
**Leukocytosis**(> 11 × 10^9/^L)							
**Yes**	17	32.1%	11	44.0%	6	21.4%	0.08
**No**	36	67.9%	14	56.0%	22	78.6%	
**Lymphocyte,** %	53	26.249 (13.17)	25	29.22 (15.61)	28	23.59 (10.11)	0.25
**Lymphopenia** (< 20%)							
**Yes**	21	39.6%	8	32.0%	13	46.4%	0.28
**No**	32	60.4%	17	68.0%	15	53.6%	
**Lymphocytosis**(> 40%)							
**Yes**	7	13.2%	6	24.0%	1	3.6%	**0.03**
**No**	46	86.8%	19	76.0%	27	96.4%	
**CRP,** mg/L	53	22.5 (5.0–82.75)	25	23.0 (3.5–81.0)	28	15.5 (4.25–60.25)	0.82
**ESR,** mm/h	16	20.0 (14.0–59.25)	5	19.0 (13.0–85.0)	11	21.0 (14.0–51.0)	0.86
**O**_**2**_ **saturation,** %	51	96.5 (94.0–97.75)	25	93.0 (86.5–95.0)	26	97.5 (95.0–98.0)	**< 0.01**
**Respiratory rate**	52	32.48 (13.36)	25	37.88 (15.66)	27	27.48 (8.35)	**0.01**
**Antiviral therapy**							
**Yes**	11	20.8%	4	16.0%	7	25.0%	0.42
**No**	42	79.2%	21	84.0%	21	75.0%	
**Antibacterial therapy**							
**Yes**	49	92.5%	23	92.0%	26	92.9%	0.91
**No**	4	7.5%	2	8.0%	2	7.1%	
**Comorbid disease**							
**Comorbid disease**							
**Yes**	20	37.7%	12	48.0%	8	28.6%	0.14
**No**	33	62.3%	13	52.0%	20	71.4%	
**G6PD deficiency**							
**Yes**	4	7.5%	3	12.0%	1	3.6%	0.25
**No**	49	92.5%	22	88.0%	27	96.4%	
**Cardiovascular**							
**Yes**	3	5.7%	2	8.0%	1	3.6%	0.49
**No**	50	94.3%	23	92.0%	27	96.4%	
**Gastrointestinal**							
**Yes**	3	5.7%	2	8.0%	1	3.6%	0.49
**No**	50	94.3%	23	92.0%	27	96.4%	
**Other comorbid diseases**							
**Yes**	6	11.3%	2	8.0%	4	14.3%	0.47
**No**	47	88.7%	23	92.0%	24	85.7%	

CRP, C-reactive protein; ESR, erythrocyte sedimentation rate. Continuous and categorical variables were analyzed using independent t-test/Mann-Whitney U test and χ2/Fisher’s exact tests, respectively. *P*-value less than 0.05 was considered as significant

**Table 2:** Comparison of clinical and laboratory features among different age groups in pediatric patients with COVID-19

	Total		Age class 1 (0–2)		Age class 2 (3–12)		Age class 3 (13–17)		P- value
	N	Mean (SD)/percentage	N	Mean (SD)/percentage	N	Mean (SD)/percentage	N	Mean (SD)/percentage	
**Severe**									
**Severe**	25	47.2%	5	71.4%	14	51.9%	6	31.6%	0.15
**Non-severe**	28	52.8%	2	28.6%	13	48.1%	13	68.4%	
**Sex**									
**Male**	22	41.5%	1	14.3%	13	48.1%	8	42.1%	0.27
**Female**	31	58.5%	6	85.7%	14	51.9%	11	57.9%	
**Disease onset to hospital admission duration**	53	4.47 (3.55)	7	4.43 (4.31)	27	4.70 (3.58)	19	4.16 (3.38)	0.89
**Hospital duration**	53	5.37 (4.22)	7	4.00 (2.16)	27	6.07 (3.94)	19	4.89 (5.08)	0.07
**ICU duration**	53	0.69 (2.46)	7	0.85 (1.57)	27	1.00 (3.26)	19	0.21 (0.91)	0.31
**ICU admission**									
**Yes**	6	11.3%	2	28.6%	3	11.1%	1	5.3%	0.25
**No**	47	88.7%	5	71.4%	24	88.9%	18	94.7%	
**Fever**									
**Yes**	42	79.2%	4	57.1%	24	88.9%	14	73.7%	0.14
**No**	11	20.8%	3	42.9%	3	11.1%	5	26.3%	
**Dry cough**									
**Yes**	40	75.5%	7	100.0%	17	63.0%	16	84.2%	0.07
**No**	13	24.5%	0	0.0%	10	37.0%	3	15.8%	
**Nasal congestion**									
**Yes**	5	9.4%	2	28.6%	3	11.1%	0	0.0%	0.08
**No**	48	90.6%	5	71.4%	24	88.9%	19	100.0%	
**Poor feeding**									
**Yes**	2	3.8%	2	28.6%	–	–	–	–	**–**
**No**	51	96.2%	5	71.4%	–	–	–	–	
**Body pain**									
**Yes**	14	30.4%	–	–	5	18.5%	9	47.4%	**0.04**
**No**	32	69.6%	–	–	22	81.5%	10	52.6%	
**Nausea**									
**Yes**	12	26.1%	–	–	8	29.6%	4	21.1%	0.51
**No**	34	73.9%	–	–	19	70.4%	15	78.9%	
**Diarrhea**									
**Yes**	5	9.4%	0	0.0%	3	11.1%	2	10.5%	0.66
**No**	48	90.6%	7	100.0%	24	88.9%	17	89.5%	
**Vomiting**									
**Yes**	11	20.8%	0	0.0%	8	29.6%	3	15.8%	0.18
**No**	42	79.2%	7	100.0%	19	70.4%	16	84.2%	
**Abdominal pain**									
**Yes**	3	6.5%	–	–	2	7.4%	1	5.3%	0.77
**No**	43	93.5%	–	–	25	92.6%	18	94.7%	
**Distress**									
**Yes**	23	43.4%	5	71.4%	13	48.1%	5	26.3%	0.09
**No**	30	56.6%	2	28.6%	14	51.9%	14	73.7%	
**Outcome**									
**Dead**	1	1.9%	0	0.0%	1	3.7%	0	0.0%	0.61
**Alive**	52	98.1%	7	100.0%	26	96.3%	19	100.0%	
**Leukocyte count,** × 10^9^/L	53	9232.08 (4755.19)	7	10,085.71 (2848.05)	27	9085.19 (5532.67)	19	9126.32 (4251.64)	0.48
**Leukopenia** (< 3.5 × 10^9^/L)									
**Yes**	3	5.7%	0	0.0%	3	11.1%	0	0.0%	0.22
**No**	50	94.3%	7	100.0%	24	88.9%	19	100.0%	
**Leukocytosis** (> 11 × 10^9/^L)									
**Yes**	17	32.1%	4	57.1%	9	33.3%	4	21.1%	0.21
**No**	36	67.9%	3	42.9%	18	66.7%	15	78.9%	
**Lymphocyte,** %	53	26.24 (13.17)	7	30.95 (12.65)	27	26.67 (13.71)	19	23.90 (12.71)	0.37
**Lymphopenia** (< 20%)									
**Yes**	21	39.6%	1	14.3%	10	37.0%	10	52.6%	0.19
**No**	32	60.4%	6	85.7%	17	63.0%	9	47.4%	
**Lymphocytosis** (> 40%)									
**Yes**	7	13.2%	2	28.6%	3	11.1%	2	10.5%	0.43
**No**	46	86.8%	5	71.4%	24	88.9%	17	89.5%	
**CRP,** mg/L	53	40.13 (45.93)	7	4.57 (5.71)	27	50.93 (45.82)	19	37.89 (48.80)	**0.01**
**ESR,** mm/h	16	34.75 (29.03)	2	22.50 (2.12)	10	46.20 (31.55)	4	12.25 (3.86)	0.05
**O**_**2**_ **saturation,** %	51	93.61 (5.89)	7	87.14 (9.70)	26	93.62 (4.99)	18	96.11 (2.90)	**0.01**
**Antiviral therapy**									
**Yes**	11	20.8%	1	14.3%	4	14.8%	6	31.6%	0.35
**No**	42	79.2%	6	85.7%	23	85.2%	13	68.4%	
**Antibacterial therapy**									
**Yes**	49	92.5%	7	100.0%	26	96.3%	16	84.2%	0.22
**No**	4	7.5%	0	0.0%	1	3.7%	3	15.8%	
**Comorbid disease**									
**Comorbid disease**									
**Yes**	20	37.7%	3	42.9%	10	37.0%	7	36.8%	0.96
**No**	33	62.3%	4	57.1%	17	63.0%	12	63.2%	
**G6PD deficiency**									
**Yes**	4	7.5%	1	14.3%	1	3.7%	2	10.5%	0.53
**No**	49	92.5%	6	85.7%	26	96.3%	17	89.5%	
**Cardiovascular**									
**Yes**	3	5.7%	2	28.6%	1	3.7%	0	0.0%	**0.02**
**No**	50	94.3%	5	71.4%	26	96.3%	19	100.0%	
**Gastrointestinal**									
**Yes**	3	5.7%	0	0.0%	2	7.4%	1	5.3%	0.75
**No**	50	94.3%	7	100.0%	25	92.6%	18	94.7%	
**Other comorbid diseases**									
**Yes**	6	11.3%	0	0.0%	3	11.1%	3	15.8%	0.53
**No**	47	88.7%	7	100.0%	24	88.9%	16	84.2%	

CRP, C-reactive protein; ESR, erythrocyte sedimentation rate. Continuous and categorical variables were analyzed using independent t-test/Mann-Whitney U test and χ2/Fisher’s exact tests, respectively. P-value less than 0.05 was considered as significant

### Clinical findings

The most common symptoms at the time of patients’ admission to hospital were fever (42 (79.2%)), followed by dry cough (40 (75.5%)). It was reported that 23 patients showed signs of respiratory distress (43.4%) in the course of their hospitalization. Generally, it was reported that the severe and non-severe groups did not differ significantly in terms of their clinical symptoms such that 19 and 23 severe patients showed fever and dry cough compared to 21 and 19 non-severe patients, respectively (*P* = 0.58 and *P* = 0.17, respectively). Similarly, dry cough and fever were the most prevalent clinical symptoms demonstrated in different age groups.

### Comorbidities, treatments and outcomes

Eleven (20.8%) and 49 (92.5%) patients received antivirals and antibiotics. Twenty (37.7%) patients showed comorbidities such that glucose-6-phosphate dehydrogenase (G6PD) deficiency (4 (7.5%)), CVD (3 (5.7%)), and gastrointestinal disorders (3 (5.7%)) were the most common ones. Twelve of the severely infected patients (12/25, 48.0%) showed underlying diseases, while a number of eight non-severe patients (8/28, 28.6%) had comorbidities (*P* = 0.14). Additionally, fifty-two patients (94.5%) showed clinical improvements after a period of two weeks (mortality rate = 5.5%). A 12-year-old patient, who showed fever, vomiting, abdominal pain, lymphopenia, high CRP (150 mg/dl), and G6PD deficiency with GGO chest finding, died from COVID-19.

### Laboratory findings

Patients with severe COVID-19 infection showed lymphocytosis, while the non-severe patients did not (*P* = 0.04). Moreover, no significant differences were observed between the severe and non-severe groups concerning CRP and ESR (*P* = 0.82 and *P* = 0.86, respectively). Furthermore, no significant differences were found among other laboratory findings between the three age-specified groups (*P* > 0.05 for all comparisons) with regard to age-specified grouping, apart from CRP, which was significantly lower in patients aged lower than two years than those with 3–12 and 13–17 years of age (*P* = 0.01),. Nevertheless, the ESR level difference between different age groups was somewhat statistically significant (*P* = 0.05).

### Chest CT findings

The findings obtained from chest CT scan were compared with regard to the disease severity and the three different age-specified groups as presented in Table 3 and Table 4, respectively. Chest CT findings were normal in 36 (67.9%) patients; moreover, severe groups (12 (48.0%)) were reported to have a higher number of abnormal CT findings than non-severe (5 (17.9%)) ones (*P* = 0.02). Moreover, GGOs (12 (22.6%)) and consolidation (10 (18.29%)), followed by subpleural sparing (5 (9.4%)), were the dominant findings in abnormal CT scans. Comparison of each CT item showed that CT findings were not significantly different between severe and non-severe infected patients (*P* > 0.05 for all the comparisons). GGO was detected in one (14.3%), six (22.2%), and five (26.3%) patients from class I, class II, and class III age groups, respectively (*P* = 0.81). Also, consolidation was reported in one (14.3%), three (11.1%), and six (28.6%) patients from class I, class II, and class III age groups, respectively (*P* = 0.21).

**Table 3:** Comparison of chest CT features between severe and non-severe pediatric patients with COVID-19

	Total		Severe		Non severe		P- value
	N	Mean (SD)/percentage	N	Mean (SD)/percentage	N	Mean (SD)/percentage	
**CT**							
**Normal**	36	67.9%	13	52.0%	23	82.1%	**0.019**
**Abnormal**	17	32.1%	12	48.0%	5	17.9%	
**Ground Glass Opacity**							
**Yes**	12	22.6%	7	28.0%	5	17.9%	0.38
**No**	41	77.4%	18	72.0%	23	82.1%	
**Peripheral halo**							
**Yes**	2	3.8%	1	4.0%	1	3.6%	0.93
**No**	51	96.2%	24	96.0%	27	96.4%	
**Consolidation**							
**Yes**	10	18.9%	7	28.0%	3	10.7%	0.11
**No**	43	81.1%	18	72.0%	25	89.3%	
**Subpleural sparing**							
**Yes**	5	9.4%	2	8.0%	3	10.7%	0.74
**No**	48	90.6%	23	92.0%	25	89.3%	

Categorical variables were analyzed using χ2/Fisher’s exact tests, respectively. P-value less than 0.05 was considered as significant

**Table 4:** Comparison of chest CT features among different age groups in pediatric patients with COVID-19

	Total		Age class I (0–2)		Age class II (3–12)		Age class II (13–17)		P- value
	N	Mean (SD)/percentage	N	Mean (SD)/percentage	N	Mean (SD)/percentage	N	Mean (SD)/percentage	
**CT**									
**Normal**	36	67.9%	6	85.7%	18	66.7%	12	63.2%	0.54
**Abnormal**	17	32.1%	1	14.3%	9	33.3%	7	36.8%	
**Ground Glass Opacity**									
**Yes**	12	22.6%	1	14.3%	6	22.2%	5	26.3%	0.81
**No**	41	77.4%	6	85.7%	21	77.8%	14	73.7%	
**Peripheral halo**									
**Yes**	2	3.8%	0	0.0%	0	0.0%	2	10.5%	0.16
**No**	51	96.2%	7	100.0%	27	100.0%	17	89.5%	
**Consolidation**									
**Yes**	10	18.9%	1	14.3%	3	11.1%	6	31.6%	0.21
**No**	43	81.1%	6	85.7%	24	88.9%	13	68.4%	
**Subpleural sparing**									
**Yes**	5	9.4%	1	14.3%	2	7.4%	2	10.5%	0.84
**No**	48	90.6%	6	85.7%	25	92.6%	17	89.5%	

Categorical variables were analyzed using χ2/Fisher’s exact tests, respectively. P-value less than 0.05 was considered as significant

## Discussion

Coronaviruses are enveloped RNA viruses from the family *Coronaviridae* that causes a variety of diseases in mammals and birds, such as human respiratory syndrome [22]. A variety of studies have revealed that pediatric patients infected with COVID-19 show a mild respiratory infection compared to the adult population [23, 24]. COVID-19 disease is of the utmost significance in children and in the physiological differences between this population and adults. Thus, this study was performed on 53 pediatric patients with RT-PCR confirmed COVID-19 who were admitted to the hospital. After their admission, their clinical, laboratory, and radiological findings were evaluated. The results showed that patients in the severe group had more respiratory distress, hospitalization and ICU duration, lymphocytosis, and lower O_2_ saturation than non-severe patients. Severe patients also showed a greater number of abnormal CT findings, particularly GGO and consolidation findings. Besides, CRP levels were normal in patients under two years of age, while it was significantly higher in both other groups which include older patients. Also, it was found that with regard to chest CT findings, GGO, and consolidation had higher frequency.

In line with the previous studies, the most commonly observed symptoms were fever and dry cough [23, 25–27], similar to other viral respiratory infections that affect children [28]. In this regard, both dry cough and fever were the most common clinical manifestations in each age group. Additionally, the findings of this study showed that a small percentage of patients were admitted to the ICU, which is consistent with findings of other studies [4, 26]. As a result, the main reported reasons are as follows: the more active the innate immune system in children at the time of exposure to a virus transmitted to them from other family members and mutated and weakened several times, the less the activity or the fewer angiotensin-converting enzyme 2 (ACE2) receptors in children. A systematic review study revealed that comorbidities with the highest frequency in children with COVID-19 were asthma, immunosuppression, and cardiovascular disease (CVD) [3], while in our study the most common comorbidities included G6PD deficiency, CVD, and gastrointestinal disease. Furthermore, regarding the different age-specified groups in this study, class I showed more cardiovascular comorbid disease than classes II and III. Besides, patients in age class II showed the highest percentages of fever and the longest hospitalization period, which implies the higher severity of this pneumonia in this age group.

Different and conflicting laboratory findings have recently been reported about the different age groups with COVID-19-confirmed patients [1, 29]. Similar to many other viral infections, the infection caused by is expected to lead to an increased number of lymphocytes, although most studies have contrarily shown a decrease in lymphocytes in these patients [21, 22, 30]. This finding suggests that one of the causes may be lymphocyte consumption. Yet, in our study, the severe group showed significant lymphocytosis, which was consistent with results found in the study by Sun et.al. on COVID-19-diagnosed infants aged lower than one year [31]. Also, in a meta-analysis conducted on a pediatric population with COVID-19, lymphocytosis, and leukopenia were regarded as the main indices for pediatric inpatients [32]. It should be noted that the stage of the disease seems to play a crucial role in how lymphopenia or lymphocytosis are developed. Generally, lymphocytosis emerges at the early stages of the disease, but at the late stages, it occurs due to lymphocyte consumption in the activation against virus and as a result of apoptosis. Therefore, it is important to pay attention to the stages of the disease in the lymphocyte count and immune cells, in general, to the extent that disregarding this issue can lead to conflicting results in various studies. In the groups formed based on the patients’ age, it was shown that as the patients’ age increased, O_2_ saturation decreased. On the other hand, nasal congestion, dry cough, respiratory distress, and disease severity were more commonly shown in the age class I than in classes II and III. The average CRP was normal in class I, but it suddenly increased in classes II and III. Therefore, it seems that CRP could not be a reliable marker for the severity of the disease in COVID-19 infant patients. Rather, it is an effective index in children aged more than two years.

This study included four children with G6PD deficiency, three of whom were placed in the severe group. Infections such as COVID-19 can trigger hemolysis of red blood cells in G6PD deficiency patients [33–35]. Wu et.al. showed that G6PD deficiency enhances human coronavirus infection in the cell culture [36]. Hydroxychloroquine, which is used as an effective drug to treat COVID-19 in many medical centers, has pro-hemolytic effects [33, 37, 38]. A number of COVID-19 patients have been reported to show hemolysis symptoms after they took hydroxychloroquine [34, 39]. However, none of the patients with G6PD deficiency in this study received hydroxychloroquine. Accordingly, it can be suggested that this drug should be used with caution in COVID-19 patients who suffer from G6PD deficiency or use any other alternative drug.

Although no specific clinical or radiologic findings are available for COVID-19 diagnosis, a chest CT scan is useful in identifying the severity of lung lesions in patients with pneumonia [40]. In the present study, approximately two-thirds of patients were presented with normal chest CT scans and demonstrated a mild, non-deteriorating course of infection. This finding is in line with those of the recent study conducted by He et.al. on 35 children with COVID-19 in Beijing [27]. Patients in the severe group showed more chest CT findings such as consolidation and GGO than the non-severe patients, though it was not statistically significant. In addition, a significant difference was seen in severe patients compared to the non-severe group. In agreement with the present study is the fact that the destruction of pulmonary parenchyma in radiological findings manifests itself as GGO and consolidation [4, 13, 41, 42]. Also, the presence of consolidation suggests the infiltration of inflammatory cells into the lungs and, consequently, damage to the pulmonary parenchyma. However, the age groups did not differ significantly in terms of their chest CT findings. All in all, the use of CT findings, especially GGO and consolidation, along with other clinical findings, can be effective in the early detection of severe COVID-19.

To the best of the authors’ knowledge, this is the first study that compares the clinical, laboratory, and CT findings of severe and non-severe COVID-19 pediatric patients among different age groups. Although the present study was conducted on a larger sample size than the similar ones performed on pediatric COVID-19 patients, it has its owm limitations. One of the limitations of this study is the small sample size, especially in the age group with patients aged less than two years. Also, this study included only a short, three-month observational design with a retrospective nature. Moreover, on the account that the obtained data came only from Iran, there was no way to make a comparison between the clinical data from US and European examinations on children with COVID-19. Notably, as an exclusion criteria, we had to exclude those patients with lack of data regarding CT findings, while those with some missing clinical/laboratory data were not excluded. Due to the prevalence of some respiratory infections in children and the similarities and overlaps between radiological findings of these infections and those caused by coronavirus, more comprehensive and epidemiological studies are needed to find differential radiologic findings between these infections. It is suggested that further studies with larger sample sizes as well as comparisons with adult populations be conducted so as to make explicit the differences in the symptoms and pathogenesis of coronavirus in the pediatric population.

## Conclusion

There is a crucial need to better recognize the full laboratory spectrum of COVID-19 in different pediatric populations in order to establish an early diagnosis of the disease. Moreover, this study is believed to be the first attempt in comparing the aforementioned findings in different age groups. Findings revealed that lymphocytosis and abnormal CT findings (GGO and consolidation) are the most reliable factors associated with COVID-19 severity. Also, it was found in this study that the severity of COVID-19 and respiratory distress decreased with age (in the group with patients aged less than 17 years).

## Data Availability

The dataset analyzed during the current study are available from the corresponding author on reasonable request.

## Abbreviations

COVID-19: Coronavirus Disease 19
SARS-CoV-2: Severe Acute Respiratory Syndrome Coronavirus 2
CT: Computed Tomography
GGO: Ground-glass Opacity
RT-PCR: Reverse Transcriptase-polymerase Chain Reaction
ICU: Intensive Care Unit
G6PD: Glucose 6 Phosphatase Dehydrogenase

## References

[1] Vakili S, Savardashtaki A, Jamalnia S, Tabrizi R, Nematollahi MH, Jafarinia M, Akbari H (2020). Laboratory findings of COVID-19 infection are conflicting in different age groups and pregnant women: a literature review. Arch Med Res.

[2] Akbari H, Tabrizi R, Lankarani KB, Aria H, Vakili S, Asadian F, et al. The role of cytokine profile and lymphocyte subsets in the severity of coronavirus disease 2019 (COVID-19): A systematic review and meta-analysis. Life Sci. 2020:118167. 10.1016/j.lfs.2020.118167 PMC7387997 32735885

[3] Patel NA (2020). Pediatric COVID-19: systematic review of the literature. Am J Otolaryngol - Head Neck Med Surg.

[4] Xia W, Shao J, Guo Y, Peng X, Li Z, Hu D (2020). Clinical and CT features in pediatric patients with COVID-19 infection: different points from adults. Pediatr Pulmonol.

[5] Ludvigsson JF (2020). Systematic review of COVID-19 in children shows milder cases and a better prognosis than adults. Acta Paediatr.

[6] Riphagen S, Gomez X, Gonzalez-Martinez C, Wilkinson N, Theocharis P (2020). Hyperinflammatory shock in children during COVID-19 pandemic. Lancet..

[7] Covid CDC, COVID CDC, COVID CDC, Bialek S, Gierke R, Hughes M (2020). Coronavirus disease 2019 in children—United States, February 12–April 2, 2020. Morb Mortal Wkly Rep.

[8] Ai T, Yang Z, Hou H, Zhan C, Chen C, Lv W, Tao Q, Sun Z, Xia L (2020). Correlation of Chest CT and RT-PCR Testing in Coronavirus Disease 2019 (COVID-19) in China: A Report of 1014 Cases. Radiological Society of North America (RSNA).

[9] Wu J, Wu X, Zeng W, Guo D, Fang Z, Chen L, Huang H, Li C (2020). Chest CT findings in patients with coronavirus disease 2019 and its relationship with clinical features. Investig Radiol.

[10] Steinberger S, Lin B, Bernheim A, Chung M, Gao Y, Xie Z, et al. CT Features of Coronavirus Disease (COVID-19) in 30 Pediatric Patients. Am J Roentgenol. 2020:1–9. 10.2214/AJR.20.23145 32442030

[11] Chung M, Bernheim A, Mei X, Zhang N, Huang M, Zeng X, Cui J, Xu W, Yang Y, Fayad ZA, Jacobi A, Li K, Li S, Shan H (2020). CT imaging features of 2019 novel coronavirus (2019-nCoV). Radiology..

[12] Duan Y, Zhu Y, Tang L, Qin J. CT features of novel coronavirus pneumonia (COVID-19) in children. Eur Radiol. 2020:1–7. 10.1007/s00330-020-06860-3 PMC7156230 32291501

[13] Li W, Cui H, Li K, Fang Y, Li S. Chest computed tomography in children with COVID-19 respiratory infection. Pediatr Radiol. 2020:1–4. 10.1007/s00247-020-04656-7 PMC7080075 32162081

[14] Shi S, Qin M, Shen B, Cai Y, Liu T, Yang F (2020). Association of Cardiac Injury With Mortality in Hospitalized Patients With COVID-19 in Wuhan, China. Association of Cardiac Injury with Mortality in hospitalized patients with COVID-19 in Wuhan.

[15] Li K, Wu J, Wu F, Guo D, Chen L, Fang Z, et al. The Clinical and Chest CT Features Associated with Severe and Critical COVID-19 Pneumonia. Invest Radiol. 2020;1. 10.1097/RLI.0000000000000672 PMC7147273 32118615

[16] National Health Commission of the People’s Republic of China. Diagnosis and treatment protocol for novel coronavirus pneumonia (6th edition). Available from http://www.nhc.gov.cn/yzygj/s7653p/202002/8334a8326dd94d329df351d7da8aefc2.shtml. [Accessed on Febr.

[17] Gong J, Dong H, Xia SQ, Huang YZ, Wang D, Zhao Y, et al. Correlation analysis between disease severity and inflammation-related parameters in patients with COVID-19 pneumonia. MedRxiv. 2020. 10.1186/s12879-020-05681-5 PMC7750784 33349241

[18] Organization WH. Paediatric age categories to be used in differentiating between listing on a model essential medicines list for children. 2014;:1–5.

[19] Zarei F, Jalli R, Iranpour P, Sefidbakht S, Soltanabadi S, Rezaee M, Jahankhah R, Manafi A Differentiation of chest CT findings between influenza pneumonia and COVID- 19 : Interobserver agreement between radiologists Objectives : To investigate the chest CT and clinical characteristics of COVID-19 pneumonia and. Acad Radiol 2021. doi:10.1016/j.acra.2021.04.010, Differentiation of Chest CT Findings Between Influenza Pneumonia and COVID-19: Interobserver Agreement Between Radiologists. 10.1016/j.acra.2021.04.010 PMC8112282 34024714

[20] Inui S, Fujikawa A, Jitsu M, Kunishima N, Watanabe S, Suzuki Y, Umeda S, Uwabe Y (2020). Chest CT findings in cases from the cruise ship “diamond princess” with coronavirus disease 2019 (COVID-19). Radiol Cardiothorac Imaging.

[21] Chen N, Zhou M, Dong X, Qu J, Gong F, Han Y, Qiu Y, Wang J, Liu Y, Wei Y, Xia J', Yu T, Zhang X, Zhang L (2020). Epidemiological and clinical characteristics of 99 cases of 2019 novel coronavirus pneumonia in Wuhan, China: a descriptive study. Lancet..

[22] Huang C, Wang Y, Li X, Ren L, Zhao J, Hu Y, Zhang L, Fan G, Xu J, Gu X, Cheng Z, Yu T, Xia J, Wei Y, Wu W, Xie X, Yin W, Li H, Liu M, Xiao Y, Gao H, Guo L, Xie J, Wang G, Jiang R, Gao Z, Jin Q, Wang J, Cao B (2020). Clinical features of patients infected with 2019 novel coronavirus in Wuhan. China Lancet.

[23] Cai J, Xu J, Lin D, Xu L, Qu Z, Zhang Y, et al. A Case Series of children with 2019 novel coronavirus infection: clinical and epidemiological features. Clin Infect Dis. 2020;:pii: ciaa198. 10.1093/cid/ciaa198 PMC7108143 32112072

[24] Shen KL, Yang YH, Jiang RM, Wang TY, Zhao DC, Jiang Y (2020). Updated diagnosis, treatment and prevention of COVID-19 in children: experts’ consensus statement (condensed version of the second edition). World J Pediatr.

[25] Bialek S, Gierke R, Hughes M, McNamara LA, Pilishvili T, Skoff T (2020). Coronavirus disease 2019 in children — United States, February 12–April 2, 2020. MMWR Morb Mortal Wkly Rep.

[26] Peng X, Xu X, Li Y, Cheng L, Zhou X, Ren B (2020). Transmission routes of 2019-nCoV and controls in dental practice. Int J Oral Sci.

[27] He M, Wang C, Xu L, Zhang H, Liu Y, Zhao Y, He S, Zhang Y, Yang H, Liu Y, Miao M, Chen Z, Pang L (2020). Epidemiological and clinical characteristics of 35 children with COVID-19 in Beijing. China Pediatr Investig.

[28] Lu CY, Huang LM, Fan TY, Cheng AL, Chang LY (2018). Incidence of respiratory viral infections and associated factors among children attending a public kindergarten in Taipei City. J Formos Med Assoc.

[29] Ghahramani S, Tabrizi R, Lankarani KB, Kashani MA, Rezaei S, Zeidi N, et al. Laboratory features in severe vs. non-severe COVID-19 patients in Asian populations, a systematic review and meta-analysis. Eur J Med Res. 2020;25. 10.1186/s40001-020-00432-3 PMC7396942 32746929

[30] Du W, Yu J, Wang H, Zhang X, Zhang S, Li Q (2020). Clinical characteristics of COVID-19 in children compared with adults in Shandong Province. China Infection.

[31] Sun D, Chen X, Li H, Lu XX, Xiao H, Zhang FR, Liu ZS (2020). SARS-CoV-2 infection in infants under 1 year of age in Wuhan City. China World J Pediatr.

[32] Ma X, Liu S, Chen L, Zhuang L, Zhang J, Xin Y. The clinical characteristics of pediatric inpatients with SARS-CoV-2 infection: A meta-analysis and systematic review. Journal of Medical Virology. 2020;:10.1002/jmv.26208, 2021. 10.1002/jmv.26208 PMC7323441 32558955

[33] Mohammad S, Clowse MEB, Eudy AM, Criscione-Schreiber LG (2018). Examination of hydroxychloroquine use and hemolytic Anemia in G6PDH-deficient patients. Arthritis Care Res.

[34] Beauverd Y, Adam Y, Assouline B, Samii K (2020). COVID-19 infection and treatment with hydroxychloroquine cause severe haemolysis crisis in a patient with glucose-6-phosphate dehydrogenase deficiency. Eur J Haematol.

[35] Afra TP, Vasudevan Nampoothiri R, Razmi TM (2020). Doubtful precipitation of hemolysis by hydroxychloroquine in glucose-6-phosphate dehydrogenase-deficient patient with COVID-19 infection. Eur J Haematol.

[36] Wu YH, Tseng CP, Cheng ML, Ho HY, Shih SR, Chiu DTY (2008). Glucose-6-phosphate dehydrogenase deficiency enhances human coronavirus 229E infection. J Infect Dis.

[37] Youngster I, Arcavi L, Schechmaster R, Akayzen Y, Popliski H, Shimonov J, Beig S, Berkovitch M (2010). Medications and glucose-6-phosphate dehydrogenase deficiency: an evidence-based review. Drug Saf.

[38] Ashley EA, Recht J, White NJ (2014). Primaquine: the risks and the benefits. Malar J.

[39] Maillart E, Leemans S, Van Noten H, Vandergraesen T, Mahadeb B, Salaouatchi MT (2020). A case report of serious haemolysis in a glucose-6-phosphate dehydrogenase-deficient COVID-19 patient receiving hydroxychloroquine. Infect Dis (Auckl).

[40] Song W, Li J, Zou N, Guan W, Pan J, Xu W (2020). Clinical features of pediatric patients with coronavirus disease (COVID-19). J Clin Virol.

[41] Chung M, Bernheim A, Mei X, Zhang N, Huang M, Zeng X, Cui J, Xu W, Yang Y, Fayad ZA, Jacobi A, Li K, Li S, Shan H (2020). CT imaging features of 2019 novel coronavirus (2019-NCoV). Radiology..

[42] Y. X, D. S, Y. L, Y. F, L. Z, X. L, et al. Clinical and High-Resolution CT Features of the COVID-19 Infection: Comparison of the Initial and Follow-up Changes. Invest Radiol. 2020. doi:10.1097/RLI.0000000000000674. 10.1097/RLI.0000000000000674 PMC7147282 32134800

